# Comparison of the efficacy of a distal clavicular locking plate with and without a suture anchor in the treatment of Neer IIb distal clavicle fractures

**DOI:** 10.1186/s12891-019-2892-6

**Published:** 2019-10-30

**Authors:** Hua Xu, Wen Jun Chen, Xiao Cheng Zhi, Shi Chang Chen

**Affiliations:** 1 Department of Orthopaedics Surgery, Shanghai Baoshan Hospital of Integrated Traditional Chinese and Western Medicine, 181 YouYiRoad, Shanghai, 201999 China; 2 Department of Orthopaedics Surgery,HuaShan hospital, Affiliated with Fudan University, 12 Middle Urumchi Road, Shanghai, 200040 China

**Keywords:** Distal clavicle fracture, Locking plate, Suture anchor, Clinical outcome

## Abstract

**Background:**

To compare the clinical outcomes between the use of a distal clavicular locking plate alone and the combined use of a plate and a coracoclavicular suture anchor in the treatment of Neer IIb distal clavicle fractures and to discuss the application procedure of suture anchors.

**Methods:**

This is a retrospective study. Thirty-four patients with unilateral Neer IIb distal clavicle fractures who underwent open reduction and internal fixation with a distal clavicular locking plate only (16 patients) or with both a plate and a coracoclavicular suture anchor (18 patients) were evaluated. The main observation data included the Constant-Murley Shoulder Function Score (CMS), rate of postoperative complications, and union time.

**Results:**

The distal clavicular locking plate and coracoclavicular suture anchor combination group had better outcomes in the Constant-Murley score (94.6 ± 4.5 vs. 90.1 ± 9.5) (*P* < 0.05) and a shorter union time (13.9 ± 2.3 vs. 16.1 ± 3.0) (P < 0.05) than the locking plate only group did, and the rate of complications showed no significant difference, 16.7% vs. 31.2% (5/16) (P>0.05).

**Conclusions:**

Both methods achieved good results in the treatment of Neer IIb distal clavicle fractures; however, the use of both locking plates and coracoclavicular suture anchors can provide more stability in the early stage after operation than can the use of locking plates alone, which can make the sped of union quicker and result in better clinical outcomes. For elderly patients with comminuted Neer IIb distal clavicle fractures, a locking plate combined with a suture anchor is recommended to provide more stability in the early stage after the operation.

## Background

Distal clavicle fractures represent nearly 20% of all clavicle fractures [1]. A Neer IIb distal clavicle fracture is a special type of injury that results in apparent displacement, and open reduction and internal fixation are often required for this type of injury [2]. Treatment of Neer IIb distal clavicle fractures is still a controversial topic for orthopaedic surgeons. To date, numerous surgical techniques for the treatment of these unstable fractures have been reported, but each procedure has its disadvantages. The combined use of a coracoclavicular suture anchor and locking plate and the use of a locking plate alone for this type of injury were both reported to achieve good clinical outcomes. Because the combined use of coracoclavicular suture anchors and locking plates costs more and causes more trauma to patients, this study preliminarily investigated the appropriate conditions for the use of a suture anchor.

## Methods

The inclusion criteria for this study were (a) acute fractures, (b) Neer IIb distal clavicle fractures, (c) internal fixation with a distal clavicular locking plate or internal fixation with both a plate and a suture anchor, and (d) normal shoulder function before injury. The exclusion criteria were (1) additional ipsilateral or contralateral upper extremity injury, (2) open fracture, and/or (3) shoulder joint arthritis detected by a preoperative examination. These criteria yielded 36 patients from 2010.2 to 2017.1 after institutional review of patients’ electronic medical records and radiographs. These patients were asked to participate in a research examination consisting of a CMS assessment and radiographic analysis. A total of 34 patients chose to participate and were included in the final analysis. All 34 patients were divided into 2 groups according to the treatment methods: 18 patients (group A) underwent fixation with a distal clavicular locking plate and a suture anchor, and 16 patients (group B) underwent fixation with a locking plate only. X-rays from the first postoperative visit to the last follow-up were analysed for loss of reduction and displacement of hardware. Functional evaluations were analysed by the CMS score.

### Surgical procedure

Surgery was performed under general anaesthesia in a semi-sitting position by two experienced doctors. One dose of intravenous cefuroxime (1.5 g) was administered to all patients 30 min before surgery. A standard anterior approach to the clavicle was used. In group A, the fracture was reduced under direct visualization, and the initial reduction was held with Kirschner wires. Then, the bottom of the coracoid process was exposed. One suture anchor (3.5-mm titanium anchor with 2 sutures, Smith & Nephew, USA) was inserted in the base of the coracoid process. Then, the distal clavicular locking plate was placed on the reduced clavicle fracture with the maximum number of locking screws incorporated into the distal fragment. The sutures were pulled through the holes or notches of the plate and several knots were tied. For group B, the procedures were the same except for the exposure of the coracoid process and the use of a suture anchor.

### Postoperative management

A shoulder arm sling was used for 3 weeks postoperatively. Passive external rotation was encouraged, and pendulum exercises began at 2 weeks. At 3 weeks, the sling was removed, and active movement of the shoulder was encouraged if no hardware complications occurred. Full return to activities and/or sports was allowed at 3 to 6 months depending on the results of the radiographs. All patients were followed up at postoperative months 1, 3, 6 and at the last follow-up and underwent clinical and radiographic evaluations that continued in 6-month intervals thereafter. All patients had the implants removed after union of the fracture occurred, which usually occurred 12 months after the initial operation. The CMS was used postoperatively after 1, 3, and 6 months and the last follow-up to assess the performance of the joint. During the follow-ups, complications including infection, malunion, and nonunion (based on the Zanca radiographic view) were evaluated. Nonunion was defined based on Neer’s original description as a “lack of bone bridging for more than 12 months after injury” [3]. Loss of reduction was evaluated in each patient by measuring and comparing the side of fracture with the opposite side on the follow-up radiographs.

### Statistical analysis

Data were analysed by using SPSS software (version 16.0, SPSS Inc., Chicago, IL, USA). Differences between the distal clavicular locking plate only group and the plate and suture anchor combination group were compared by using an independent t-test and χ^2^ test. Statistical significance was defined as 0.05, and a *p*-value< 0.05 was taken to indicate a statistically significant difference.

## Results

Thirty-four patients with Neer IIb clavicle fractures were included in our retrospective study. A summary of the patients’ profiles is shown in Table 1. Both groups were similar with respect to age, gender, and confounding medical conditions (*P* > 0.05). The distal clavicular locking plate and suture anchor combination group (group A) included 18 patients with an average age of 45.5 years; 9 patients suffered from a high-trauma fracture (6 patients had vehicular trauma, 3 patients had a fall from height), and 9 patients suffered from a low-trauma fracture (a fall) [4]. The distal clavicular locking plate only group (group B) included 16 patients with an average age of 50.7 years; 9 patients suffered from a high- trauma fracture (6 patients had vehicular trauma, 3 patients had a fall from height), and 7 patients suffered from a low-trauma fracture (a fall).

**Table 1:** Demographics of 34 patients

Variables	Number of patients	Age	Gender		Injury type			Fracture side		Duration to operation
–	–	–	Male	Female	Fall	vehicular trauma	Fall from height	Left	Right	(d, x ± s)
Group A	18	45.5 ± 14.4	12	6	9	6	3	11	7	5.1 ± 2.3
Group B	16	50.7 ± 17.7	9	7	7	6	3	8	8	5.5 ± 2.6
F value	–	−0.944	0.073		0.133			0.093		−0.476
*P* value	–	0.352	0.787		0.936			0.760		0.637

A *p*-value< 0.05 was taken to indicate a statistically significant difference

**Fig. 1:**
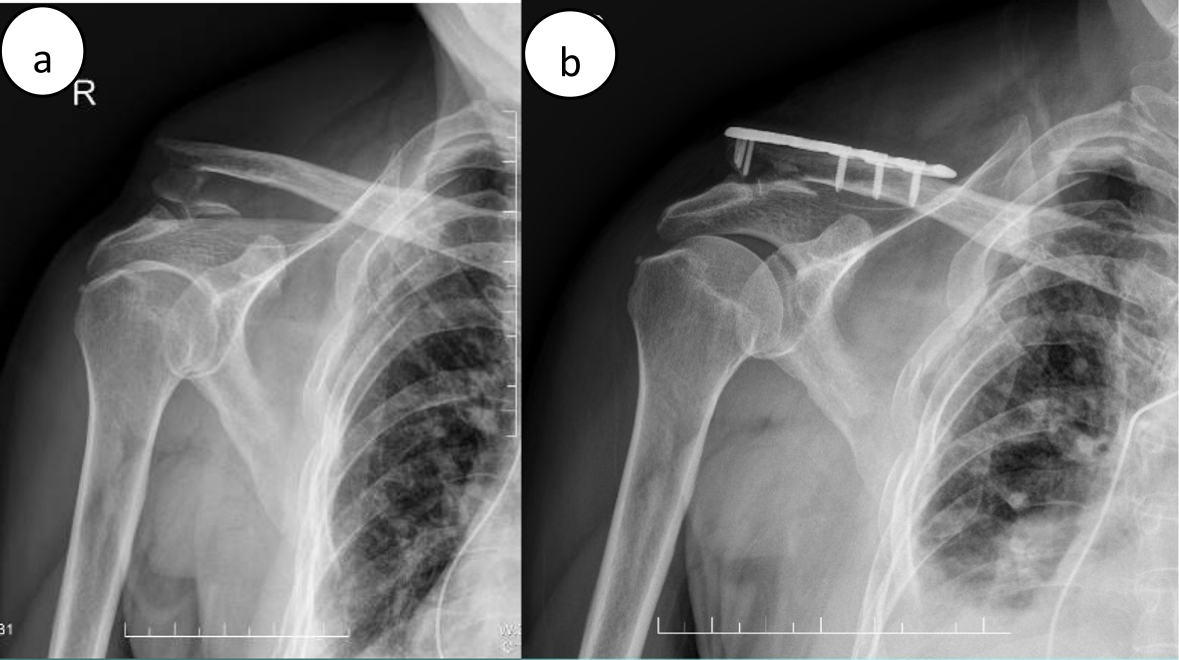
**a** . X-ray of a patient before surgery. **b**. Post-operation x-ray showed loss of reduction

The average clinical follow-up times were 17.5 and 16.0 months in groups A and B, respectively (*P* > 0.05). The CMS score of group A at 1 month/3 months/6 months postoperatively and the last follow-up were 82~96/82~98/82~98/82~100, respectively, and the scores of group B were 69~91/71~96/71~98/71~100, respectively (*P* < 0.05). The mean time of bone union was 13.9 ± 2.3 weeks and 16.1 ± 3.0 weeks in groups A and B, respectively (*P* > 0.05). In group A, two patients complained of implant-related discomfort during shoulder motion, and another patient experienced delayed union of the fracture. In group B, 3 out of the 5 patients with complications showed delayed union, one of whom showed loss of reduction, which was healed by retardation (Fig. 1). One of the remaining two patients complained of implant-related discomfort during shoulder motion, and the other patient had superficial wound infection, which was healed after the administration of antibiotics. Nonunion was not observed, and no screws or plates were broken. The mean time of implant removal was 12 months (11 to 14 months) in both groups (*P* > 0.05). In total, complications occurred in 3 patients in group A (16.7%) and in 5 patients in group B (31.2%), which was not a significant difference (*P* > 0.05). Table 2 shows the details of the clinical results.

**Table 2:** Comparison of clinical results

	Number of patients	Constant score of last follow up (points)				postoperative Constant score (points)			Union time (week)	Complication rate
		Pain	ADL	Mobility	Strength	1month	3months	6 months		
Group A	18	13.9 ± 2.1	18.9 ± 1.2	37.7 ± 2.1	24.2 ± 2.0	90.1 ± 3.8	93 ± 3.9	93.4 ± 4.0	13.9 ± 2.3	16.7%
Group B	16	12.5 ± 4.1	18.6 ± 1.6	35.5 ± 3.5	23.4 ± 2.4	84.2 ± 7.4	87.5 ± 8.9	87.9 ± 9.2	16.1 ± 3.0	31.2%
F value	–	1.230	0.623	2.189	1.060	2.871	2.285	2.213	−2.415	0.355
*P* value	–	0.232	0.538	0.039	0.297	0.009	0.033	0.039	0.022	0.551

A *p*-value< 0.05 was taken to indicate a statistically significant difference

## Discussion

The Neer IIb pattern comprises a fracture between the coracoclavicular ligaments, and the typical presentation of the injury is complete rupture of the coracoclavicular ligament [3]. These fractures can be significantly displaced because the trapezius muscle pulls the medial fragment superiorly and posteriorly and the weight of the arm on the shoulder girdle pulls the distal fragment inferiorly and anteriorly. As a result, the nonunion rate is reported to be between 25 and 44% with non-operative management [5]. Surgical management of Neer IIb fractures is advocated by Neer and other authors. Many implants and surgical methods are reported for patients who need surgery, including Kirschner wires, coracoclavicular screws, hook plate fixation, flexible coracoclavicular fixation, and locking plate fixation. However, all of these methods have disadvantages. Fixation with intramedullary K-wire is a simple procedure [6], but many researchers do not propose it because of complications including pin migration, infections caused by the pin, and failure of fixation [7, 8]. Patients treated with coracoclavicular screws need screw removal, and complications such as the screw backing out and mal-union occur in these patients [9, 10]. Closed-loop double endo-button fixation is a popular flexible coracoclavicular technology for distal clavicle fractures; however, the length of the loop is not easy to control. In addition, some of the patients developed frozen shoulder and asymptomatic nonunion [10, 11]. Hook plate has been proven to be an effective treatment for many years, but a second surgery to remove the hardware to prevent sub-acromial impingement and rotator cuff tears is essential [12, 13]. In addition, fixation with a hook plate is associated with acromial fractures in patients with osteoporosis [14].

The use of locking plates only and the combined use of the plates and suture anchors are both reported to be effective in the treatment of distal clavicle fractures because of fewer complications compared with the use of hook plates [15]. The combination approach using an anchor leads to more trauma to expose the coracoid process and is more costly than the use of a locking plate only; thus, in which condition it is necessary to use coracoclavicular augmentation (including suture anchor fixation)? There is no exact answer for this question. It has been reported that mal-union, loss of reduction and nonunion occurred in Raju Vaishya’s cases treated with locking plates alone [16]. From our experience, it is not easy to place a sufficient number of screws in the distal shaft for comminuted fractures, especially in older patients with low bone mineral density. Loss of reduction and delayed union occurred in patients with comminuted fractures in group B. This result indicates that the use of a locking plate alone is not sufficiently stable for some cases of Neer IIb distal clavicle fractures. A cadaveric study revealed that the suture anchor was as strong as the coracoclavicular ligament and could replace the coracoclavicular ligament [17]. Placing a suture anchor from the coracoid to the clavicle is reported to be an effective method because the anchor plays the role of the coracoclavicular ligament. Klein SM [18] reported the method of using both a locking plate and a suture anchor and considered the method to be a good method.

We acknowledge that there are several limitations in this study. First, this was a retrospective study and was not randomized. Second, the cohort was relatively small. The “post hoc” power of χ^2^ test used in this study was 0.417, which revealed the possible influence of type 2 errors, indicating actually we may even under-estimated the difference in the current study. Up to now, nearly all the literatures published about Neer IIb distal clavicle fractures had limitations of small number. Third, the follow-up period was relatively short. Base on the finding of this study, the study group is planning to launch another prospective, multi-centered, randomized clinical trial, and recruit the patients in that study to further validate this conclusion.

## Conclusions

Both methods achieved good results in the treatment of Neer IIb distal clavicle fractures; however, the use of both a locking plate and a coracoclavicular suture anchor can provide more stability in the early stage after operation than the use of a locking plate alone can, which can make the union quicker and result in better clinical outcomes. However, the use of both the plate and anchor costs more and causes more trauma to patients than the use of the plate only. For elderly patients with comminuted Neer IIb distal clavicle fractures, a locking plate combined with a suture anchor is recommended to provide more stability in the early stage after the operation.

## Data Availability

All data generated or analyzed during this study are included in this published article. Hua Xu and ShiChang Chen can be contacted to request the raw data.

## Abbreviations

CMS Score: Constant-Murley Shoulder Function Score.
CT: Computed tomography.
